# Different Approaches to the Treatment of Anterior Tooth Fractures: Three Clinical Cases and Behavior Report

**DOI:** 10.7759/cureus.64524

**Published:** 2024-07-14

**Authors:** Denitsa Zaneva-Hristova, Tsvetelina Borisova-Papancheva, Yanko G Yankov, Georgi Papanchev

**Affiliations:** 1 Department of Conservative Dental Treatment and Oral Pathology, Medical University "Prof. Dr. Paraskev Stoyanov", Varna, BGR; 2 Clinic of Maxillofacial Surgery, University Hospital St. Marina, Varna, BGR; 3 Department of General and Operative Surgery, Medical University "Prof. Dr. Paraskev Stoyanov", Varna, BGR; 4 Department of Oral Surgery, Medical University "Prof. Dr. Paraskev Stoyanov", Varna, BGR

**Keywords:** freehand technique, milk, saline, silicone key, fractured fragment, uncomplicated fracture, andreasen's classification, dental injuries, dental trauma, tooth fracture

## Abstract

Fractures of the anterior teeth are a common form of dental trauma. This article includes three case reports of uncomplicated fractures of upper anterior teeth in which collaborators had different treatment protocols. The choice of the treatment method is based on the direction of the specific clinical case and the clinical findings. Of great importance to the treatment approach are the measures taken by the patient to preserve the fractured fragment, the age of the fracture, and the time available to patients and clinicians. When the fractured fragment is available and is well-preserved, the best approach is to fix it to the crown of the tooth. This protocol is extremely fast and inexpensive, with minimal potential for problems in esthetics and function. In the absence of the fractured fragment, the treatment approaches are different, as described in cases 2 and 3. If the patient or the clinician is unable to make a repeat visit, the restoration is carried out using a freehand technique. Protocols involving fracture repair using composite materials are more labor-intensive. Esthetic complications are often observed, which may be due to wrong determined shade, loss of luster, and change over the years in the color of the restoration, as well as fracture of the restoration. With advances in dentistry, these disadvantages have been minimized.

## Introduction

Tooth fractures are common in children and young patients, accounting for 5% of all injuries [1]. Around 25% of all school-age children experience trauma to the hard dental tissues, and 33% of adults experience trauma to the permanent dentin [1]. Tooth fractures can result from sports accidents, car accidents, or falls [1]. Crown fracture of maxillary anterior teeth is the most common type of dental trauma due to its location in the dental arch [2]. Crown fractures of permanent incisors account for about 18-22% of dental hard tissue injuries, among which 28-44% are uncomplicated and 11-15% are complicated fractures [2]. As a result, there are limitations in function, esthetics, and phonetics [2]. Fractures of the front teeth are most often seen in men in the age group between 36 and 45 years [3].

The most commonly preferred treatment protocol for such cases is the use of an esthetic composite restoration to restore the lost tooth structure [4]. An overbite and overjet of more than 3 mm has been found to be one of the causes that lead to tooth fracture [5]. Most patients with trauma to an anterior tooth have an Angle class 1 occlusion [6].

Over the years, traumatic dental injuries have been diagnosed and reported according to various factors such as anatomy, etiology, pathology, therapeutic approach, and degree of severity. The variety of diagnostic methods used complicates the accurate documentation of traumatic dental injuries and can lead to serious consequences, medico-legal problems, and reduced quality of life for patients.

Since 1955, many classifications of traumatic dental injuries have been developed and proposed, such as Sweet's classification (1955), Spina's classification (2002), Galea's classification (1984), Bennett's classification (1963), Ellis' classification (1970), Andreasen's classification (1981), and many others [7].

Andreasen's classification has become a global standard for clinicians and researchers over the years. It is closely related to the specific tissue injuries, treatment methods, and later complications of dental injuries. Andreasen's widely recognized classification of traumatic dental injuries was fully adopted by the WHO in 2022 [8].

Andreasen's classification (1981) divides trauma into four groups: trauma to the hard dental tissues and pulp, trauma to the periodontal tissues, trauma to the underlying bone, and trauma to the gums or oral mucosa [7]. We will consider the classification of the first group since it is within the scope of our study. It classifies fractures according to the structures they involve. The first three subgroups include disorders of the crown. The first subgroup is an incomplete fracture (crack) of the enamel without loss of tooth substance. In the second subgroup are all fractures involving enamel or involving enamel and dentin but not exposing the pulp. A third subgroup is of complicated fractures that involve enamel and dentin with pulp involvement. The next subgroup includes all uncomplicated crown and root fractures that involve enamel, dentin, and cementum but do not involve the pulp. The fifth subgroup includes all complicated crown and root fractures, including enamel, dentin, cementum, and pulp. The last subgroup includes root fractures.

This report presents already-known but important information that, through these three clinical cases, enriches the current medical literature by sharing our experience with the treatment of these patients.

## Case presentation

Clinical case 1

**Figure 1:**
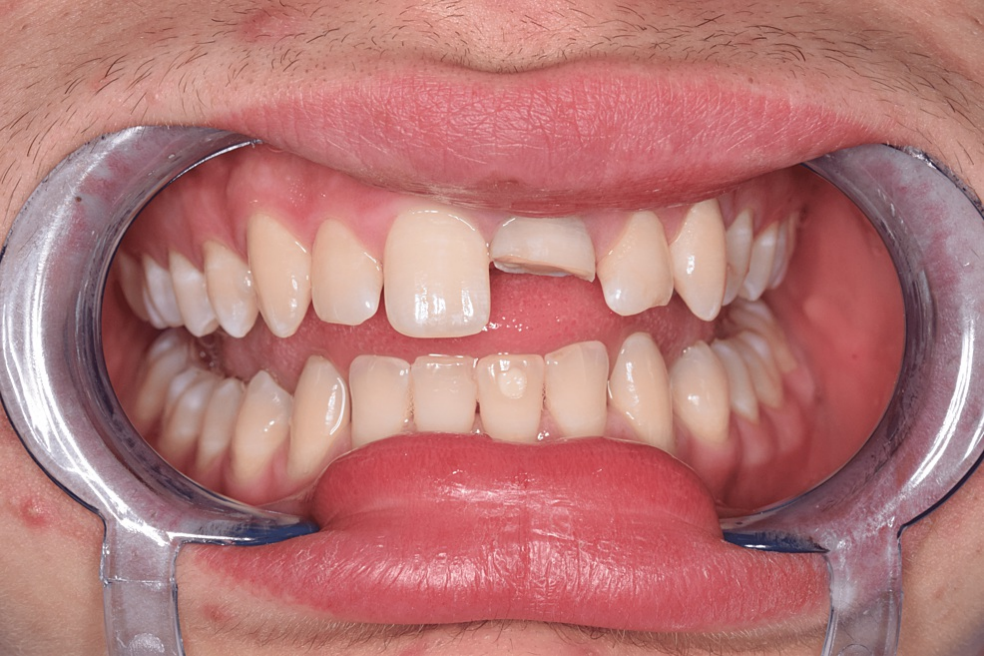
Clinical case 1: Uncomplicated fracture of tooth 21 (class 2)

A 22-year-old young man consulted by telephone with specialists in conservative dentistry at the Medical-Dental Center at Varna Medical University, reporting a sports accident in April 2023, as a result of which a part of his central upper incisor was fractured. The patient was instructed to store the fractured fragment in saline and was given a clinic appointment. On the same day, a full clinical examination of the patient was performed and routine paraclinical tests were assigned (pulp test, periapical radiographs). An uncomplicated horizontal fracture of tooth 21 was found, covering more than 1/3 of the clinical crown, but not affecting the pulp chamber. According to the Andreasen classification, it was a class 2 fracture (Figure 1).

**Figure 2:**
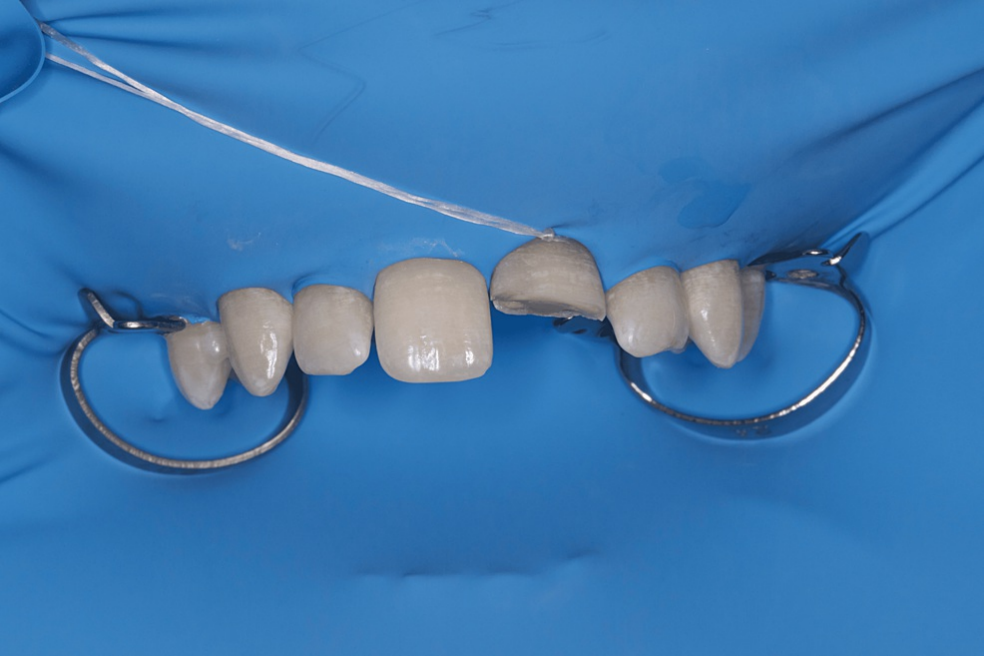
Clinical case 1: Isolation of the operative field with a rubber dam

Since the patient successfully preserved the fractured fragment, we were able to fix it on the tooth. To isolate the operative field, we used a rubber dam, isolating teeth 14 to 24 with winged premolar clamps (No. 208). To further retract the dam in the area of ​​tooth 21, we used a single ligature of dental floss (Figure 2).

Adjacent teeth 11 and 22 were isolated using Teflon tape with a thickness of 0.2 mm. In the area of ​​the fracture line, the tooth was medicated with 2% chlorhexidine gluconate and then rinsed abundantly with distilled water. We switched to selective etching with 39% phosphoric acid (Tokuyama Etching Gel HV) for 20 seconds. The fractured fragment was also etched with the same etching gel. The next step involved applying an adhesive material along the fracture line of the tooth and the fragment (Bond Force II). Using an air jet, the bond was spread and polymerized for 20 seconds. A flowable composite material (Estelite Universal Flow High, Tokuyama) was applied to the incisal part of the tooth, after which the fragment was fixed with the help of an applicator. We proceeded to photopolymerization for 40 seconds.

**Figure 3:**
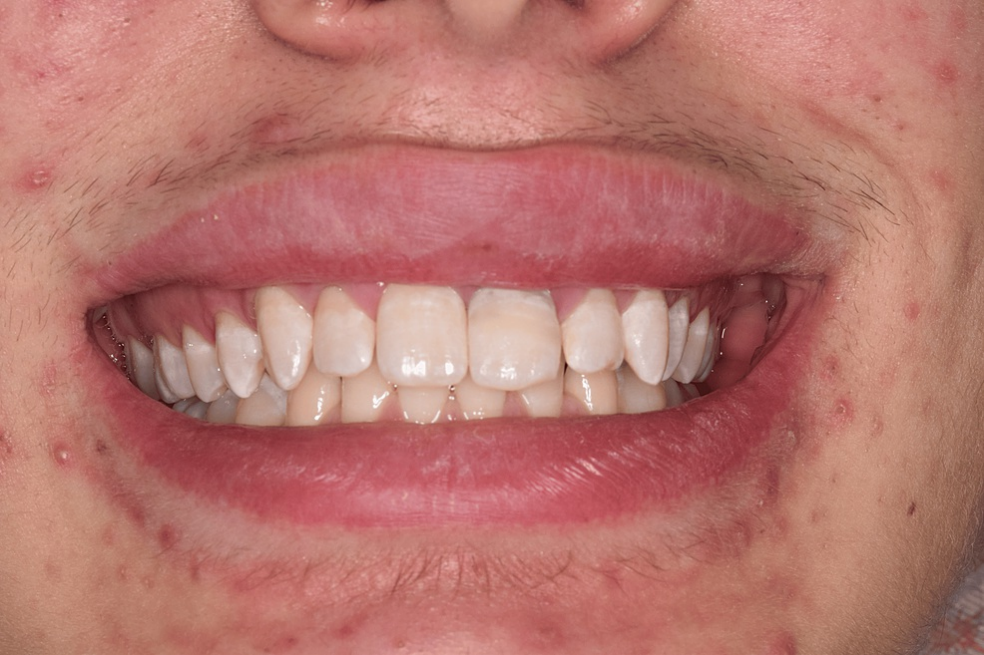
Clinical case 1: The fixed fragment of tooth 21 immediately after removal of the rubber dam

Excess composite material was removed using finishing diamond files and polishing was carried out using polishing rubbers (EVE Diacomp), brushes, and polishing paste (EVE Diacomp Paste) (Figure 3).

The patient was scheduled for a follow-up examination after six weeks, in May of the same year, to determine whether complications had occurred. No complications were found. Normal tooth color had returned. Again, we confirmed the correct occlusion. The patient reported that he is satisfied with the result.

Clinical case 2

**Figure 4:**
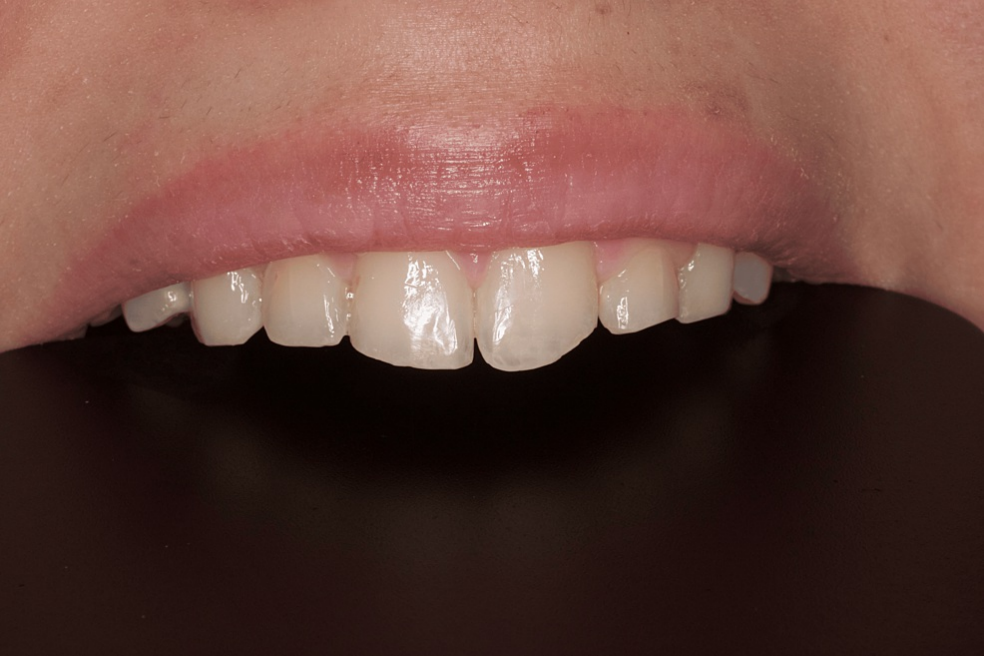
Clinical case 2: Fracture of the distal enamel edge of tooth 21 (class 1)

A 25-year-old patient came to us in June 2023 at the Medical-Dental Center at Varna Medical University with a complaint of impaired esthetics due to a fracture of the distal enamel edge of tooth 21, class 1, which was about a month old (Figure 4).

Due to the absence of other complaints, it was decided to restore the distal edge using a silicone key.

**Figure 5:**
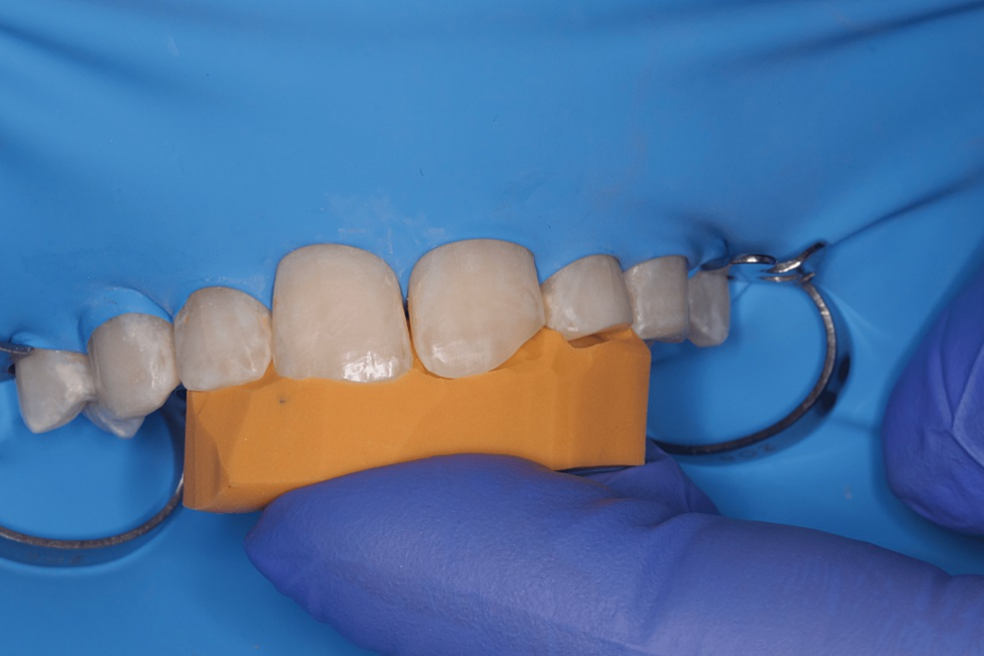
Clinical case 2: Isolation of the operative field and adaptation of the silicone key

At the first visit in June 2023, we took an impression from which the dental laboratory cast a model and modeled the fractured distal incisal edge. In the second clinical visit, five days after the first visit, we took a silicone key from the palatal and incisal part of the front teeth, and using a scalpel, we removed the excess that would prevent visual control and adequate application of the composite material on the palatal edge of the fracture and the silicone key (Figure 5).

**Figure 6:**
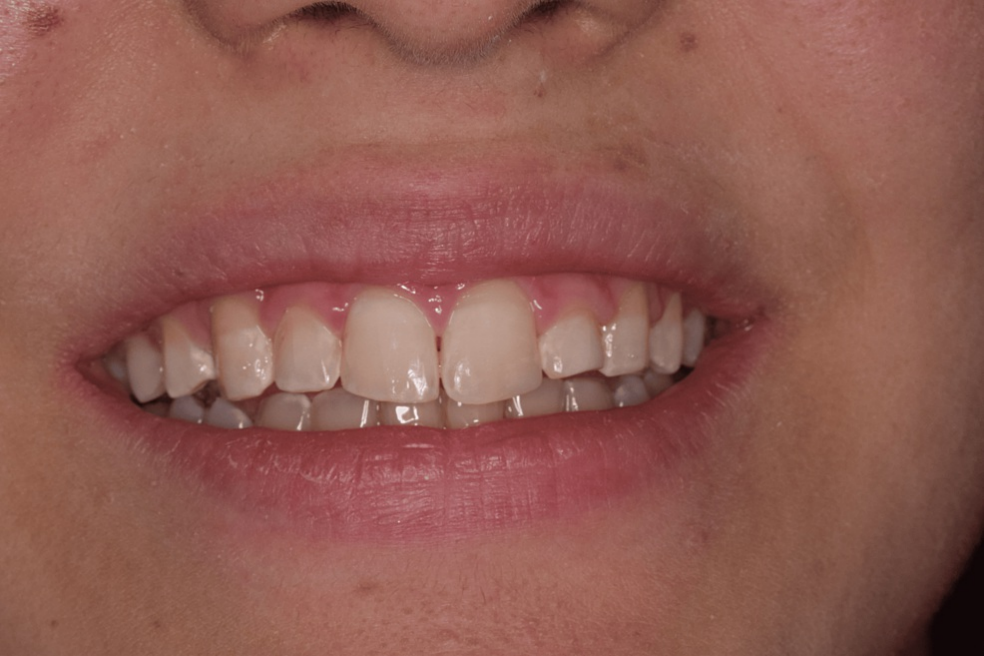
Clinical case 2: Restoration of tooth 21

We proceeded to choose a suitable shade, even before the isolation, so that the choice of the right shade would not be compromised due to the dehydration of the teeth. To isolate the operative field, we used a rubber dam, isolating teeth 14 to 24 with winged premolar clamps (No. 208). Adjacent teeth 11 and 22 were isolated using Teflon tape with a thickness of 0.2 mm. In the area of ​​the fracture line, the tooth was medicated with 2% chlorhexidine gluconate and then rinsed abundantly with distilled water. We switched to selective etching with 39% phosphoric acid (Tokuyama Etching Gel HV) for 20 seconds. The next step involved applying an adhesive material (Bond Force II). Using an air jet, the bond was spread and polymerized for 20 seconds. With the help of the silicone key, we constructed the palatal surface of the fractured tooth with a nanohybrid composite material (Tokuyama, Estelite Asteria). Using a sector metal matrix, we built the proximal wall. Finally, we completed the restoration by building the vestibular wall and the incisal edge. Excess composite material was removed using finishing diamond files and polishing was carried out using polishing rubbers (EVE Diacomp), brushes, and polishing paste (EVE Diacomp Paste) (Figure 6).

In July of the same year, the patient returned to us with another complaint. We analyzed the restoration of tooth 21 and found that the tooth was functioning normally. The teeth have regained their normal color and this has greatly improved the esthetics.

Clinical case 3

**Figure 7:**
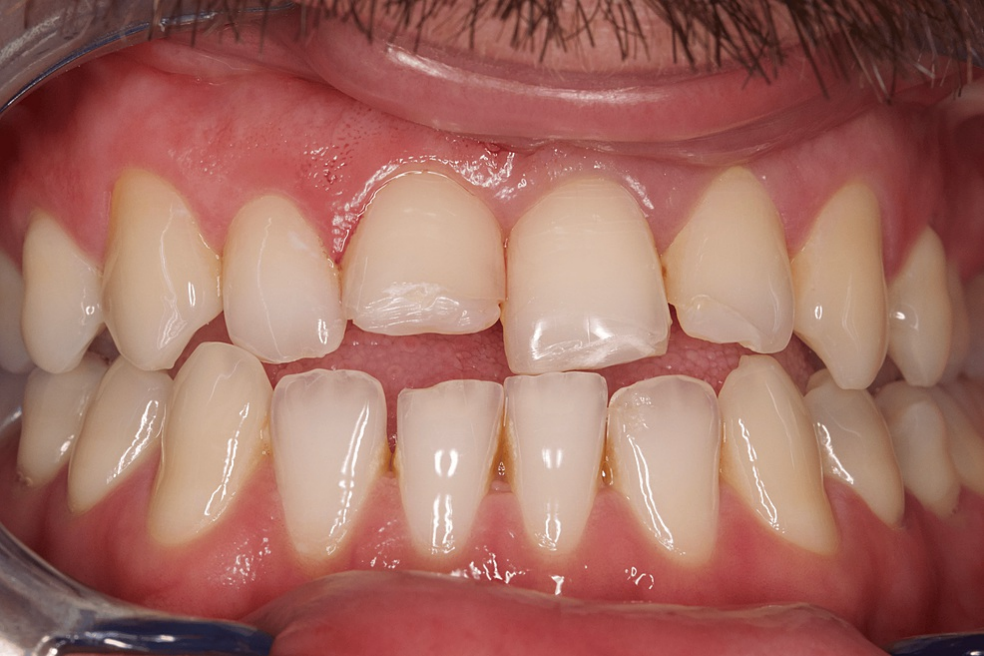
Clinical case 3: Uncomplicated fractures of teeth 11, 21, and 22 - (class 1 and 2)

A 29-year-old man suffered a sports accident in January 2024, as a result of which uncomplicated fractures of teeth 11, 21, and 22 were observed. He came to us at the Medical-Dental Center at Varna Medical University two days after the accident (Figure 7).

**Figure 8:**
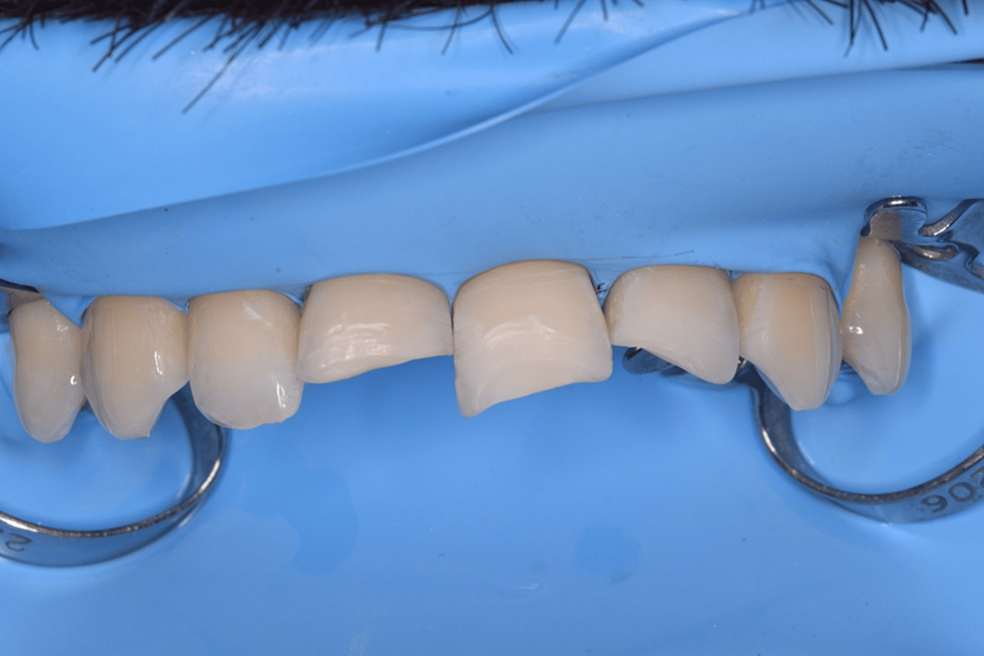
Clinical case 3: Isolation of the operative field with a rubber dam

On tooth 11, the fracture was class 2, and on the other teeth, it was class 1. The patient reported an upcoming trip and a lack of time for a second clinical visit. For this reason, we decided to restore the teeth with the freehand technique. We proceeded to choose a suitable shade (A2B, A3B, NE), even before the isolation, so that the choice of the right shade would not be compromised due to the dehydration of the teeth. To isolate the operative field, we used a rubber dam, isolating teeth 14 to 24 with winged premolar clamps (No. 208) (Figure 8).

**Figure 9:**
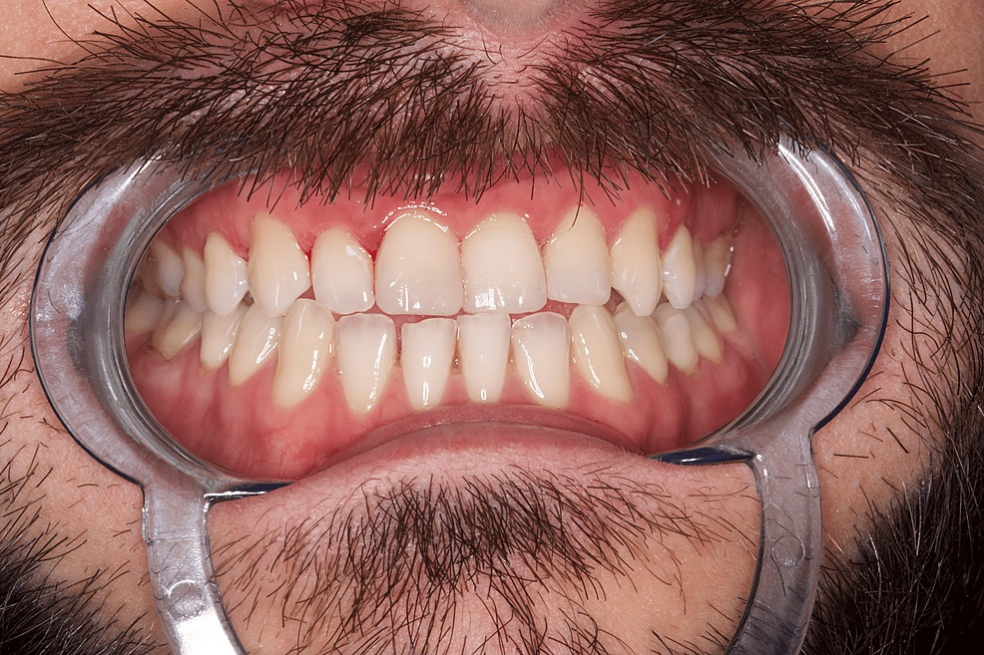
Clinical case 3: Esthetic restorations after freehand technique of teeth 11, 21, and 22

Adjacent teeth 12 and 23 were isolated using Teflon tape with a thickness of 0.2 mm. In the area of ​​the fracture line, the teeth were medicated with 2% chlorhexidine gluconate and then rinsed abundantly with distilled water. We switched to selective etching with 39% phosphoric acid (Tokuyama Etching Gel HV) for 20 seconds. The next step involved applying an adhesive material (Bond Force II). Using an air jet, the bond was spread and polymerized for 20 seconds. We constructed the palatal surfaces of the fractured teeth with a nanohybrid composite material (Tokuyama, Estelite Asteria). Using a Polydentia Unica Anterior metal matrix, we built the approximal surfaces. Finally, we completed the restoration by building the vestibular walls and incisal edges. Excess composite material was removed using finishing diamond files and polishing was carried out using polishing rubbers (EVE Diacomp), brushes, and polishing paste (EVE Diacomp Paste) (Figure 9).

We analyzed the restorations of teeth 11, 21, and 22 and found that the teeth performed normal function. The patient was satisfied with the esthetic result and reported no tension during masticatory movements.

## Discussion

When following up on the cases treated by three methods, all three gave good esthetic and functional results. Fixation of the fractured fragment should be the clinician's and patient's first choice of treatment. This is only achieved in cases where the fragment is available and has been preserved for a short period of time in a solution that does not allow its dehydration. The advantages of this protocol are that the patient's stay in the office is much less, compared to the assistance of direct restorations. Another advantage is that the esthetics and shape of the tooth that was before the time of the fracture is achieved. In this method, a small amount of composite material is used and its shrinkage is less, compared to cases where the missing part is completely restored with the composite. The disadvantage of direct restorations with a silicone key is that the treatment time is longer and more expensive. Its advantage before using the freehand technique is that the patient has previously seen the formats of the tooth, and, if necessary, corrections are made to the model.

An effective anterior fracture treatment plan not only improves function and appearance but also benefits the patient psychologically. The most preferred method of treating a fractured tooth is to restore it using the fractured fragment. This is possible only in cases where the fragment is properly stored and transported [9]. The tooth can be transported in saline or milk [9]. Milk is a good option for storing the fragment because it contains calcium and phosphate, which make the dentin surface hard and hydrated, allowing for better bond strength [9]. Another suggested storage option for the fragments is egg white, which also shows a strong bond between the fragment and the clinical dental crown [10]. If we cannot obtain either, it is possible to leave the fragment in the oral cavity and saliva. These approaches prevent fragment collapse and dehydration, thus avoiding any change in fragment sizes [11].

According to a study by Shirani et al., as of 2011, the best storage media are milk and saliva [12]. They confirm that in these two environments, small osmotic changes occur in the dentin surface and a stronger bond is achieved [12].

Intact healthy dentin that is stored in a dry environment for 24 hours retains only about 25% of the total amount of moisture. This partial loss of dentin moisture and shrinkage results in a weak bond between the composite and the dentin [13].

Reattachment of the fragment to the tooth is one of the best methods of treatment for such a condition. It is considered a better treatment option as it is uncomplicated, esthetic, cheaper, and preserves the tooth structure. The disadvantage of this method is that even after standing in one of the solutions listed above, the fragment dehydrates and loses its real color. However, one month after its fixation, the fragment was hydrated and the color returned, achieving satisfactory esthetics and function [14].

## Conclusions

There are many factors on which the approach to tooth fracture treatment depends. The clinical approach to the treatment of dental tissue fractures depends on individual cases. It is essential whether the fragment is preserved, how it is transported to the cabinet, and whether it is possible to limit the load on this section. To achieve a positive prognosis, the patient's cooperation and awareness of the treatment are essential. The presented three clinical cases confirm the conditions listed above. Good functional and esthetic qualities of the restoration were achieved with all three restoration techniques.
